# Supplementary material for: Differences in the clinical presentation of sleep apnea patients according to age and gender
Source: PLoS One. 2025 Feb 26;20(2):e0318569. doi: 10.1371/journal.pone.0318569 (PMC11864517; doi:10.1371/journal.pone.0318569)
Supplement: S1 Table — (DOCX) [file pone.0318569.s001.docx]

|  | Age-group | | |
| --- | --- | --- | --- |
|  | <70 | 70-80 | >80 |
| Men | 845 (34.3%)* | 114(29.6%)* | 47 (42.7%) |
| Women | 509 (45.0%)* | 125(43.0%)* | 46 (54.8%) |

S1 Table
