# Supplementary material for: Differences in the clinical presentation of sleep apnea patients according to age and gender
Source: PLoS One. 2025 Feb 26;20(2):e0318569. doi: 10.1371/journal.pone.0318569 (PMC11864517; doi:10.1371/journal.pone.0318569)
Supplement: S2 Table — (DOCX) [file pone.0318569.s002.docx]

S2 Table

|  | Age-group | | |
| --- | --- | --- | --- |
|  | <70 | 70-80 | >80 |
| Men | 499 (24.5%)* | 71(22.5%)* | 34 (32.1%)* |
| Women | 367 (38.3%)* | 103(41.0%)* | 37 (46.8%)* |
